# Impact of HPV vaccination on cervical screening performance: a population-based cohort study

**DOI:** 10.1038/s41416-020-0850-6

**Published:** 2020-05-04

**Authors:** Jiayao Lei, Alexander Ploner, Matti Lehtinen, Pär Sparén, Joakim Dillner, K. Miriam Elfström

**Affiliations:** 1 Department of Medical Epidemiology and Biostatistics, Karolinska Institutet, SE-171 77 Stockholm, Sweden; 2 Faculty of Social Sciences, University of Tampere, SE-330 14 Tampere, Finland; 3 Department of Laboratory Medicine, Karolinska Institutet, SE-141 83 Stockholm, Sweden; 4 Karolinska University Laboratory, Karolinska University Hospital, SE-171 76 Stockholm, Sweden; 5 Regional Cancer Center Stockholm-Gotland, SE-118 27 Stockholm, Sweden

**Keywords:** Epidemiology, Cancer

## Abstract

**Background:**

Human papillomavirus (HPV) vaccination is predicted to lower the positive predictive value (PPV) of cytology.

**Methods:**

We included 153,250 girls born between 1989 and 1993, resident in Sweden since the introduction of HPV vaccines (October 2006) and attending cervical screening at age 23 years. We assessed their first cytology and following histopathological diagnosis using Swedish National Cervical Screening Registry (NKCx). By linkage with the national Swedish HPV vaccination registry, we determined PPV of abnormal cytology for cervical intraepithelial neoplasia grade 2 or worse (CIN2+) and the differences with 95% confidence intervals (CIs) according to vaccination status.

**Results:**

The PPV of high-grade cytology for CIN2+ was 69.9% (95% CI, 67.9–71.9), 64.9% (95% CI, 59.8–69.8) and 57.4% (95% CI, 50.9–63.7) among women unvaccinated, initiating vaccination at age 17–22 years and initiating vaccination before age 17 years, corresponding to reduction in PPV by 8% (95% CI, 0–15%) and 17% (95% CI, 7–26%) in vaccinated groups after adjustment for birth cohort, respectively.

**Conclusion:**

The PPV of cytology for CIN2+ decreased among vaccinated women, and the decrease was stronger for girls vaccinated at younger ages. A switch from cytology to HPV testing might potentially improve the screening performance.

## Background

High-risk human papillomavirus (HPV) is the major cause of cervical cancer.^1^ As of October 2018, 91 countries had introduced HPV vaccines in their national immunisation programmes.^2^ In Sweden, HPV vaccines were introduced in late 2006, and starting from May 2007, HPV vaccination was subsidised for girls aged 13–17 years (birth cohorts 1989–1993). In 2012, a free-of-charge catch-up HPV vaccination programme for girls aged 13–18 years (birth cohorts 1993–1998), and a school-based HPV vaccination programme for girls aged 10–12 years (birth cohorts 1999 onwards) was launched.^3^ The effectiveness of HPV vaccine against genital warts and cervical intraepithelial neoplasia grade 2 or worse (CIN2+) observed in Swedish population^4–6^ was in line with a recent meta-analysis,^7^ and the effectiveness increases with younger age at initiating vaccination. Strongest effectiveness has been seen in girls initiating vaccination before age 17 years, with 64% effectiveness against CIN2+.^4,5^ Vaccination also provides some cross-protection against HPV types not included in the vaccines.^8–10^

According to European Union screening guidelines, women below age 30 years should be screened with cytology^11^ due to the high prevalence of HPV in this age group. In Sweden, cytology screening is recommended for women aged 23–29 years, and primary HPV screening is recommended for women aged 30 years and above. The positive predictive value (PPV) of cytology depends on the prevalence of cervical lesions and has been predicted to decline in vaccinated populations.^12^ As high PPV is essential for achieving a favourable balance between health gains in detecting high-grade cervical lesions and adverse outcomes such as unnecessary referrals, such a decline of PPV could impact screening policies for young women. The aim of our study was to evaluate whether HPV vaccination does indeed affect the PPV of abnormal cytology for CIN2+.

## Methods

### Study population

**Fig. 1:**
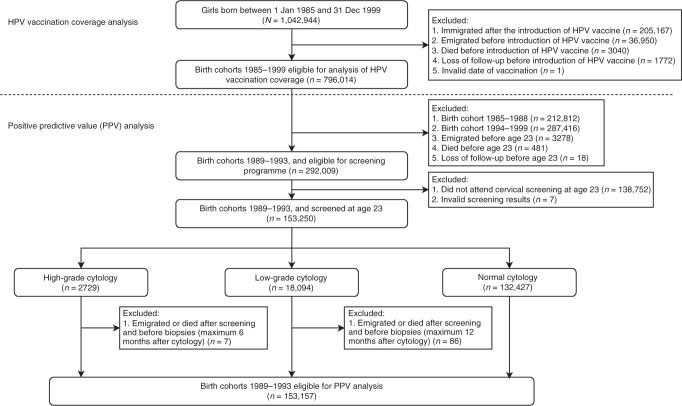
Study population.

The identities of all women born between 1 January 1985 and 31 December 1999 were retrieved from the Total Population Register (Fig. 1).^13^ Women who immigrated to Sweden after the introduction of HPV vaccines (1 October 2006), women who emigrated, died or were lost to follow-up before the introduction of HPV vaccines and women who had invalid date of vaccination were excluded, leaving the rest of the birth cohorts eligible for the analysis of HPV vaccination coverage.

For the analysis of detection rates and PPV of cytology for CIN2+, we included women born between 1 January 1989 and 31 December 1993 for whom the vaccination was available through either the subsidised opportunistic programme or the free-of-charge catch-up programme. In these birth cohorts, we identified women who attended cervical screening at age 23 years through linkage with the Swedish National Cervical Screening Registry (NKCx).^14^ As invitations to the organised cervical screening programme in Sweden start at age 23 years, women were categorised based on their first cytology result at age 23 years as having high-grade abnormal cytology, low-grade abnormal cytology or normal cytology. Women who attended cervical screening at age 23 years and who had an abnormal cytology were followed up for histopathological assessment. We excluded women who died or who emigrated after abnormal cytology and within the required interval for histopathological assessment (6 months for high-grade cytology and 12 months for low-grade cytology, according to national guidelines at the time).

### Data collection

Data were collected from the Swedish national population-based registers. All eligible women were linked through the Swedish personal identification number.^15^

The main exposure was defined as HPV vaccination based on a linkage to HPV vaccination records through 31 December 2017, and women with at least one dose of HPV vaccine before age 23 years were considered as vaccinated, otherwise as unvaccinated. Age at vaccination initiation was calculated, and categorised as age <17 and 17–22 years based on our previous work on vaccine effectiveness against HPV-related outcomes.^4,5,16^ HPV vaccination information was extracted from the Swedish HPV Vaccination Register, an informed consent-based register in operation since the introduction of HPV vaccination, and complemented with data from the Prescribed Drug Register (PDR),^17^ which is a mandatory register on dispensed prescriptions, in operation since July 2005. Both bivalent (1.3%) and quadrivalent (98.7%) HPV vaccine prescriptions were identified using Anatomical Therapeutic Chemical codes.

The outcome was high-grade cervical lesions (CIN2+) based on histopathological diagnoses from the NKCx, which contains all cytological and histopathological records since 1995 for all women in Sweden, from both the organised screening programme and opportunistic screening.^14^ Systematised Nomenclature of Medicine codes were used to define the cytological and histopathological diagnoses (Supplementary Table S1). Abnormal cytology was defined as cytological diagnoses of atypical squamous cells of undetermined significance (ASCUS) or worse. Abnormal cytology was further classified as high grade and low grade based on the severity of the cytological diagnoses. Low grade included the cytological diagnoses of ASCUS and low-grade squamous intraepithelial lesion, while the rest of the abnormal cytological diagnoses were classified as high grade, including atypical glandular cells, atypia in cells of unclear origin and CIN2+/adenocarcinoma in situ or worse.

### Statistical analysis

We estimated HPV vaccination coverage for birth cohorts 1985–1999 in total as well as by age at HPV vaccination initiation. The detection rate of high-grade cervical lesions was estimated as the proportion of women with an abnormal cytology, followed by a histopathologically confirmed CIN2+ among all women who attended screening. The PPV of cytological screening for high-grade cervical lesions was estimated as the proportion of women with an abnormal cytology and histopathologically confirmed CIN2+ among all women who had an abnormal cytology at screening. Both detection rates and PPVs were estimated separately for women with low- and high-grade cytology, considering the different clinical management strategies. All proportions were reported with 95% binomial confidence intervals (CIs). We used the log-binominal regression to estimate the ratio of PPV for CIN2+ in vaccinated women compared to unvaccinated women as risk ratio (RR), both crude and adjusted for birth cohort. Percentage change of PPV in vaccinated women was calculated as (1 − RR) × 100%.

We performed sensitivity analyses to assess the validity of our material and the robustness of our results. First, we examined the reduction of CIN2+ among vaccinated women compared to unvaccinated women, as a comparison with other vaccinated cohorts. We also calculated the PPV for CIN2+ based on the first cytology test taken at any age up to 23 years, including opportunistic tests prior to screening age, to quantify the impact of opportunistic screening. In order to assess the effect of the incomplete follow-up of histopathological diagnoses for the 1993 birth cohort, we artificially applied such a truncation for all birth cohorts. Furthermore, we examined the role of referral to histopathological assessment after abnormal cytology using a histopathological diagnosis on record as a proxy for attendance at follow-up and tabulating this with vaccination status and type of abnormal cytology. Subsequently, we re-estimated the PPV of CIN2+ limited to women who had both an abnormal cytology and a follow-up histopathological diagnosis on record.

All statistical tests were two sided. SAS 9.4 was used for data management and statistical analysis. This study was approved by the Regional Ethical Review Board in Stockholm, Sweden, which determined that written informed consent by the study participants was not required.

## Results

### Study population and vaccine coverage

**Fig. 2:**
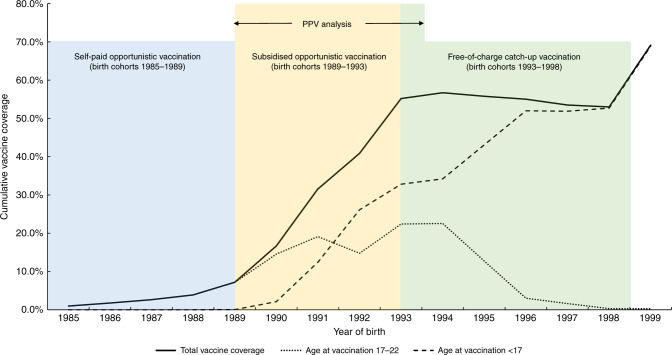
Vaccination coverage for birth cohort 1985–1999, stratified by age at vaccination initiation. *PPV*, positive predictive value.

We included 796,014 women born between 1985 and 1999, resident in Sweden since the introduction of HPV vaccination (1 October 2006) and age 23 years (Fig. 1). The total HPV vaccination coverage was below 10% for birth cohorts vaccinated through self-paid opportunistic vaccination (1985–1988). In birth cohorts eligible for subsidised opportunistic vaccination (1989–1992), the total vaccine coverage increased substantially and the coverage was ~55% among birth cohorts vaccinated through the free-of-charge, organised catch-up programme (1993–1998). The proportion of women initiating vaccination before age 17 years increased steadily by birth cohort and reached 69% in the 1999 birth cohort (Fig. 2).

Among birth cohorts 1989–1993, a total of 153,250 women attended screening at age 23 years with valid screening results, including 2729 (1.8%) women with high-grade cytology, 18,094 (11.8%) women with low-grade cytology and 132,427 (86.4%) women with a normal cytological result (Fig. 1).

### Detection rate of CIN2+

The overall detection rate of histopathologically confirmed CIN2+ after abnormal cytology was 3.8%, 2.3% and 1.5% for women unvaccinated, initiating vaccination at age 17–22 years and initiating vaccination before age 17 years. Stratifying by cytological results, the detection rate of CIN2+ after a high-grade cytology was 1.5% (95% CI, 1.4–1.5), 0.9% (95% CI, 0.8–1.0) and 0.5% (95% CI, 0.5–0.6) for women unvaccinated, initiating vaccination at age 17–22 years and initiating vaccination before age 17 years. The complementary detection rate for CIN2+ after low-grade cytology were 2.3% (95% CI, 2.2–2.4), 1.4% (95% CI, 1.3–1.5) and 1.0% (95% CI, 0.9–1.0), respectively (Table 1).

**Table 1:** Detection rate, PPV of cytology and RRs for CIN2+, in relation to age at vaccination initiation.

Cytological results	Age at vaccination initiation	Screened	Screen positive	CIN2+	Detection rate of CIN2+, % (95% CI)^a^	PPV		
		*n*	*n*	*n*		PPV for CIN2+, % (95% CI)^b^	Crude RR (95% CI)	Adjusted^c^ RR (95% CI)
High-grade cytology								
	Unvaccinated	100,400	2110	1475	1.5 (1.4–1.5)	69.9 (67.9–71.9)	Reference	Reference
	Vaccinated at age 17–22 years	26,892	368	239	0.9 (0.8–1.0)	64.9 (59.8–69.8)	0.93 (0.86–1.01)	0.92 (0.85–1.00)
	Vaccinated at age <17 years	25,865	244	140	0.5 (0.5–0.6)	57.4 (50.9–63.7)	0.82 (0.73–0.92)	0.83 (0.74–0.93)
Low-grade cytology								
	Unvaccinated	100,400	12,293	2325	2.3 (2.2–2.4)	18.9 (18.2–19.6)	Reference	Reference
	Vaccinated at age 17–22 years	26,892	2940	377	1.4 (1.3–1.5)	12.8 (11.6–14.1)	0.68 (0.61–0.75)	0.72 (0.65–0.80)
	Vaccinated at age <17 years	25,865	2775	258	1.0 (0.9–1.0)	9.3 (8.2–10.4)	0.49 (0.44–0.56)	0.56 (0.49–0.63)

*PPV* positive predictive value, *RR* risk ratio, *CIN2+* cervical intraepithelial neoplasia grade 2 or worse.

^a^Detection rate = no. of women confirmed as CIN2+/no. of women screened × 100%.

^b^PPV = no. of women confirmed as CIN2+/no. of women screen positive × 100%.

^c^Adjusted for birth cohort.

### PPV of cytology for CIN2+

The PPV of high-grade cytology for CIN2+ was 69.9% (95% CI, 67.9–71.9), 64.9% (95% CI, 59.8–69.8) and 57.4% (95% CI, 50.9–63.7) for women unvaccinated, initiating vaccination at age 17–22 years and initiating vaccination before age 17 years, respectively. After adjustment for birth cohorts, this corresponds to a reduction of PPV by 8% (RR 0.92, 95% CI, 0.85–1.00) and 17% (RR 0.83, 95% CI, 0.74–0.93) among women initiating vaccination at age 17–22 years and women initiating vaccination before age 17 years, compared to unvaccinated women.

The PPVs of low-grade cytology for CIN2+ were 18.9% (95% CI, 18.2–19.6), 12.8% (95% CI, 11.6–14.1), and 9.3% (95% CI, 8.2–10.4) for women unvaccinated, women initiating vaccination at age 17–22 years and women initiating vaccination before age 17 years. Decline of PPV was observed among women initiating vaccination at age 17–22 years with 28% (RR 0.72, 95% CI, 0.65–0.80), and women initiating vaccination before age 17 years with 44% (RR 0.56, 95% CI, 0.49–0.63) compared to unvaccinated women, after adjustment for birth cohorts.

### Sensitivity analyses

In the sensitivity analysis, we found 39% and 58% reduction of CIN2+ among women initiating vaccination at age 17–22 years and initiating vaccination before age 17 years, respectively, compared to unvaccinated women after adjustment for birth cohort (Supplementary Table S2). Calculating the PPV for CIN2+ based on the first cytology test up to age ≤23 years gave the same results as the main analysis based on the first organised screening test at age 23 years (Supplementary Table S3). After applying a data truncation for all birth cohorts, we found the decline of PPV to be robust (Supplementary Table S4). Around 90% of women with high-grade cytology in our study population had a histopathological assessment within the specified 6-month time period (Supplementary Table S5). Restricting analysis to only women with both abnormal cytology and histopathological diagnosis yielded PPV estimates comparable to our main estimates (Supplementary Table S6).

## Discussion

### Main findings and interpretations

In this large, population-based study, we found the PPV of cytology for CIN2+ after both high- and low-grade cytology decreased significantly for vaccinated compared to unvaccinated women. The reduction of PPV was observed among birth cohorts vaccinated through either subsidised opportunistic programme or the free-of-charge catch-up programme. A stronger decrease of PPV was seen for women initiating vaccination at younger ages. The decreases of PPV corresponds to the reduced detection rates of CIN2+ in HPV-vaccinated compared to unvaccinated women, and similarly the reduction was stronger for women initiating vaccination earlier.

Franco et al.^12,18^ predicted that declines in HPV infections after HPV vaccination and subsequent reduced prevalence of cervical lesions in population would inevitably lead to a decline in the PPV of cytology among HPV-vaccinated birth cohorts. We confirm the prediction^12,18^ using real-life data that the decline of the PPV is related to the strength of protection that vaccination confers. Our findings also highlighted the PPV of cytology in HPV-vaccinated birth cohorts does not only depend on vaccination coverage but also on the proportion of individuals vaccinated at younger ages.

### Detection rate of CIN2+

Lower detection rate of CIN2+ was observed in vaccinated women, with a more pronounced reduction among girls who initiate vaccination early (before age 17 years) compared to unvaccinated women. The reduction of CIN2+ among vaccinated women in our study were within the range of effectiveness of HPV vaccine against CIN2+ in other countries or regions based on an earlier systematic review.^19^ Moreover, a latest meta-analysis performed by Drolet et al.^7^ showed that HPV vaccination can effectively reduce CIN2+ by 51% and 31% in screened girls aged 15–19 and 20–24 years in post-vaccination era, respectively.

### PPV of cytology for CIN2+

We found a more pronounced decline of PPV in vaccinated women after low-grade cytology compared to high-grade cytology. This could be explained by the fact that since 2015, women with low-grade cytology have been followed up with HPV-reflex testing rather than direct referral to histopathological assessment.^20^ This policy change has affected from birth cohort 1992 onwards, resulting in a smaller proportion of women with low-grade cytology being referred to histopathological assessment, and therefore, a lower PPV for CIN2+ after low-grade cytology.

Compared to other studies, a population-based Scottish study showed significantly lower (16%) PPV of high-grade dyskaryosis for CIN2+ in HPV-vaccinated women compared to unvaccinated women, based on birth cohorts vaccinated through the free-of-charge catch-up programme.^21^ Another Scottish study found a lower but not statistically significant decreased PPV of colposcopy for CIN2+ in vaccinated (66.7%) compared to unvaccinated women (74%) in a hospital-based study.^22^ In an ecological study from Australia, a decreasing trend in PPV of cervical cytology was shown in both women aged <20 and 20–24 years after the implementation of the HPV vaccination programme.^23^ Besides, a simulation study based on Danish data also suggested that the PPV of liquid-based cytology for CIN2+ declined after HPV vaccination among samples positive for any abnormalities in screening.^24^

As overall vaccination coverage in Sweden has been stable, and the proportion of women initiating vaccination before age 17 years has increased steadily from birth cohort 1993 onwards (Fig. 2), we expect the PPV of cytology for CIN2+ to continue to decrease towards the level of PPV for women initiating vaccination before age 17 years. With a high coverage of HPV vaccination through the school-based programme (vaccine coverage of over 80%) and vaccination at younger ages (10–12 years),^25^ both direct protection from HPV vaccines and indirect protection gained from herd effects^26^ will result in very low prevalence of cervical lesions in the population. The PPV of cytology will undoubtedly reach lower levels when those birth cohorts enter the organised cervical screening in year 2022.

According to the modelling results from Franco et al.,^12,18^ when the prevalence of cervical abnormality goes below 1%, the PPV falls under 10% for the assumed levels of sensitivity. Additionally, a lower prevalence of abnormalities in cytology is likely to affect how cytologists read slides from largely vaccinated populations.^12^ This might potentially require change of screening methods in vaccinated cohorts, such as HPV screening. Up to now, evidence on comparing screening performance of cytology screening and HPV screening in HPV-vaccinated cohorts is sparse. In a follow-up study of the Compass pilot randomised trial,^27^ an increased detection of CIN2+ in highly HPV-vaccinated birth cohorts by using HPV primary screening compared to using cytology was observed, and the referral rate for colposcopy was not higher.

### Limitations and strengths of this study

We did not have complete follow-up of histopathological diagnoses for the 1993 birth cohort due to right truncation of our data. Second, non-attendance to histopathological assessment following an abnormal cytology could potentially influence the PPV. However, overall 96% of women with high-grade cytology have a histopathological assessment within 1 year,^14^ and we also observed a high attendance to histopathological assessment in our study population. Furthermore, results from sensitivity analyses supported the robustness of our estimates even when accounting for the above-mentioned limitations (Supplementary Tables S2–S9). Finally, a small proportion of HPV-vaccinated women in birth cohort 1993 (corresponding to 6.2% of vaccination doses in that birth cohort) could be misclassified as unvaccinated due to incomplete vaccination registration, even if the incompleteness of HPV registration was to a large extent supplemented by further linkages to the PDR. However, the misclassifications will result in an underestimation of the detection rate of CIN2+ and the PPV for unvaccinated women, with limited impact on estimates for vaccinated women, meaning that the results will be attenuated.

Our study investigated the PPV of cytology for CIN2+ based on the population-based, organised screening programme with individual linkage to HPV vaccination status in a nationwide setting. As the PPV is strongly influenced by the management of low-grade lesions, our strategy to evaluate the PPV by low- and high-grade cytology separately limits the bias in PPV comparisons. Allowing a different timeframe for women with low- and high-grade cytology to attend histopathological assessment reflects the clinical management for different severity of abnormalities in cytology. Moreover, cytological tests taken before age 23 years might represent a different risk profile, so only considering the first cytology taken at age 23 years as outcome could minimise possible selection bias. Individual level linkages to high-quality Swedish registries ensured identification of exposure and outcome measures, while minimising misclassification. Besides, the cervical screening data routinely collected through NKCx provides complete coverage of cytological and histopathological diagnoses from both organised screening and opportunistic screening.^14^ Finally, reporting percentage changes of PPV enhances the generalisability of our findings. It offers other countries or regions with comparable HPV vaccination and cervical screening programmes to estimate their expected level of PPV among HPV-vaccinated birth cohorts based on current level of PPV, vaccination coverage and age at vaccination initiation.

### Public health relevance and future research

Our study provides direct evidence that the PPV of abnormal cytology for CIN2+ declines with HPV vaccination, particularly when initiated at young ages. The findings should be generalisable to other countries or regions with effective HPV vaccination and organised cervical screening programmes. As additional birth cohorts with higher HPV vaccination coverage enter the screening programmes in the coming years, the PPV is likely to decline further.

The change of PPV for CIN2+ after low-grade cytology also highlights the importance of pragmatically adapting the screening programme to an increasing proportion of low-grade cytology, with low cancer risk. A reduced prevalence of HPV16/18 is confirmed by an earlier study, indicating that most of the cervical intraepithelial neoplasia among vaccinated cohorts were associated with non-vaccine HPV types.^28^ Therefore, HPV16/18 positivity in vaccinated birth cohorts entering the screening programme will most likely be an indication of persistent infections, for example, if vaccination was initiated after prior exposure to HPV. This suggests that either decreased intensity and/or switch to primary HPV screening with partial HPV genotyping should be contemplated.

In the future, surveillance of HPV infections among HPV-vaccinated cohorts and additional comparative effectiveness studies on the PPV of cytology and HPV testing for high-grade cervical lesions are needed. This could further demonstrate the optimal screening method for HPV-vaccinated birth cohorts with corresponding age of starting screening and screening intervals. Additionally, a re-evaluation of screening performance when birth cohorts vaccinated with 9-valent vaccine enter the screening programme would also be warranted.

## Conclusion

HPV-vaccinated women had a decreased PPV of cytology for CIN2+ compared to unvaccinated women. The PPV of cytology for CIN2+ also showed a stronger decrease among women initiating vaccination before age 17 years compared to women initiating vaccination at age 17–22 years relative to unvaccinated women. The performance of cytology-based screening is expected to further decline in cohorts with higher vaccination coverage, potentially impacting the choice of optimal screening strategies for younger women. Primary HPV screening with partial genotyping could be an alternative screening method for HPV-vaccinated cohorts.

## Supplementary information

Supplementary material

## Data Availability

Data from the study is available on request from corresponding author.
