# Supplementary material for: Impact of HPV vaccination on cervical screening performance: a population-based cohort study
Source: Br J Cancer. 2020 May 4;123(1):155–60. doi: 10.1038/s41416-020-0850-6 (PMC7341799; doi:10.1038/s41416-020-0850-6)
Supplement: Supplementary file 1 — Supplementary material [file 41416_2020_850_MOESM1_ESM.docx]

# **Supplementary**

## **Table S1 Systematized Nomenclature of Medicine (SNOMED) codes for cytological and histopathological diagnoses.**

| **Outcome** | | **SNOMED code** |
| --- | --- | --- |
| **Normal cytological diagnoses** | | |
|  | Benign sample | M00110 |
| **Abnormal cytological diagnoses** | | |
| Low-grade cytology | | |
|  | Atypical squamous cell of undetermined significance (ASCUS) | M69710 |
|  | Mild dysplasia/Cervical intraepithelial neoplasia (CIN) 1 | M74006 |
| High-grade cytology | | |
|  | Atypia in cells of uncertain origin | M69700 |
|  | Atypical glandular cells (AGC) | M69720 |
|  | Moderate dysplasia/Cervical intraepithelial neoplasia (CIN) 2 | M74007 |
|  | Severe dysplasia/Cervical intraepithelial neoplasia (CIN) 3/Cancer in situ (CIS) | M80702 |
|  | Suspected high-grade dysplasia (ASC-H) | M69719 |
|  | Squamous cell carcinoma | M80703 |
|  | Adenocarcinoma/Adenocarcinoma in situ (AIS) | M81403 |
| **Histopathological diagnoses of CIN2+** | |  |
|  | Cervical intraepithelial neoplasia grade 2 | M74007 |
|  | Cervical intraepithelial neoplasia grade 3 | M80702 |
|  | Carcinoma in situ | M80762 |
|  | Adenocarcinoma in situ | M81402 |
|  | Adenosquamous cell cancer in situ | M85602 |
|  | Invasive cervical cancer of any origin (squamous cell carcinoma, adenocarcinoma, adenosquamous cell carcinoma, small cell carcinoma) | M80703, M81401, M81403, M85601,  M85603, M80413 |

## **Table S2 Risk ratios (RRs) of cervical intraepithelial neoplasia grade 2 or worse (CIN2+) in relation to age at vaccination initiation.**

| **Cytological results** | **Age at vaccination initiation** | **Screened** | **Screen-positive and confirmed as CIN2+** | **Risk ratios (95% CI)** | | **VE** |
| --- | --- | --- | --- | --- | --- | --- |
|  |  | **n** | **n** | **Crude** | **Birth-cohort- adjusted** |  |
| **All** | |  |  |  |  |  |
|  | unvaccinated | 100 400 | 3800 | Reference | Reference |  |
|  | vaccinated at age 17-22 | 26 892 | 616 | 0.61 (0.56 to 0.66) | 0.61 (0.56 to 0.67) | 39% |
|  | vaccinated at age <17 | 25 865 | 398 | 0.41 (0.37 to 0.45) | 0.42 (0.37 to 0.46) | 58% |
| **High-grade cytology** | |  |  |  |  |  |
|  | unvaccinated | 100 400 | 1475 | Reference | Reference |  |
|  | vaccinated at age 17-22 | 26 892 | 239 | 0.60 (0.53 to 0.69) | 0.58 (0.50 to 0.66) | 42% |
|  | vaccinated at age <17 | 25 865 | 140 | 0.37 (0.31 to 0.44) | 0.33 (0.28 to 0.40) | 67% |
| **Low-grade cytology** | |  |  |  |  |  |
|  | unvaccinated | 100 400 | 2325 | Reference | Reference |  |
|  | vaccinated at age 17-22 | 26 892 | 377 | 0.61 (0.54 to 0.67) | 0.63 (0.57 to 0.71) | 37% |
|  | vaccinated at age <17 | 25 865 | 258 | 0.43 (0.38 to 0.49) | 0.48 (0.42 to 0.55) | 52% |

VE, vaccine effectiveness. VE= (1-adjusted RR)*100%

## **Table S3 Positive predictive value (PPV) of cytology for cervical intraepithelial neoplasia grade 2 or worse (CIN2+), in relation to age at vaccination initiation.**

| **Cytological results** | **Age at vaccination initiation** | **PPV for CIN2+, %**  **(95% CI)** | |
| --- | --- | --- | --- |
|  |  | **First cytology at age 23** | **First cytology at age ≤23** |
| High-grade cytology | |  |  |
|  | unvaccinated | 69.9 (67.9 to 71.9) | 69.3 (67.3 to 71.2) |
|  | vaccinated at age 17-22 | 64.9 (59.8 to 69.8) | 64.5 (59.6 to 69.2) |
|  | vaccinated at age <17 | 57.4 (50.9 to 63.7) | 58.5 (52.1 to 64.8) |
| Low-grade cytology | |  |  |
|  | unvaccinated | 18.9 (18.2 to 19.6) | 18.4 (17.8 to 19.1) |
|  | vaccinated at age 17-22 | 12.8 (11.6 to 14.1) | 13.3 (12.2 to 14.5) |
|  | vaccinated at age <17 | 9.3 (8.2 to 10.4) | 9.5 (8.5 to 10.5) |

## **Table S4 Detection rate, positive predictive value (PPV) of cytology and relative risks (RRs) for cervical intraepithelial neoplasia grade 2 or worse (CIN2+), in relation to age at vaccination initiation (with truncation of histopathological diagnoses at age 24).**

| **Cytological results** | **Age at vaccination initiation** | **Screened** | **Screen-positive** | **CIN2+** | **Detection rate of CIN2+, %**  **(95% CI)^a^** | **PPV** | | |
| --- | --- | --- | --- | --- | --- | --- | --- | --- |
|  |  | n | n | n |  | **PPV for CIN2+, %**  **(95% CI)^b^** | **Crude RR** | **Adjusted^c^ RR** |
| High-grade cytology | |  |  |  |  |  |  |  |
|  | unvaccinated | 100 400 | 2110 | 1290 | 1.3 (1.2 to 1.4) | 61.1 (59.0 to 63.2) | Reference | Reference |
|  | vaccinated at age 17-22 | 26 892 | 368 | 212 | 0.8 (0.7 to 0.9) | 57.6 (52.4 to 62.7) | 0.94 (0.86 to 1.04) | 0.92 (0.84 to 1.02) |
|  | vaccinated at age <17 | 25 865 | 244 | 127 | 0.5 (0.4 to 0.6) | 52.0 (45.6 to 58.5) | 0.85 (0.75 to 0.96) | 0.83 (0.73 to 0.95) |
| Low-grade cytology | |  |  |  |  |  |  |  |
|  | unvaccinated | 100 400 | 12 293 | 1644 | 1.6 (1.6 to 1.7) | 13.4 (12.8 to 14.0) | Reference | Reference |
|  | vaccinated at age 17-22 | 26 892 | 2940 | 278 | 1.0 (0.9 to 1.0) | 9.5 (8.4 to 10.6) | 0.71 (0.63 to 0.80) | 0.76 (0.67 to 0.86) |
|  | vaccinated at age <17 | 25 865 | 2775 | 187 | 0.7 (0.6 to 0.8) | 6.7 (5.8 to 7.7) | 0.50 (0.44 to 0.58) | 0.59 (0.50 to 0.69) |

1. Detection rate = No. of women confirmed as CIN2+ / No. of women screened * 100%
2. PPV = No. of women confirmed as CIN2+ / No. of women screen-positive * 100%
3. Adjusted for birth cohort

## **Table S5 Attendance of biopsy after abnormal cytology.**

| **Cytological results^a^** | **Age at vaccination initiation** | **Screen-positive**  **n** | **Biopsy**  **n (%)** |
| --- | --- | --- | --- |
| High-grade cytology | |  |  |
|  | unvaccinated | 2110 | 1909 (90.5) |
|  | vaccinated at age 17-22 | 368 | 327 (88.9) |
|  | vaccinated at age <17 | 244 | 217 (88.9) |
| Low-grade cytology | |  |  |
|  | unvaccinated | 12 293 | 7610 (61.9) |
|  | vaccinated at age 17-22 | 2940 | 1614 (54.9) |
|  | vaccinated at age <17 | 2775 | 1222 (44.0) |

## **Table S6 Positive predictive value (PPV) of cytology and relative risks (RRs) for cervical intraepithelial neoplasia grade 2 or worse (CIN2+), in relation to age at vaccination initiation among women had both cytology and histopathological diagnoses.**

| **Cytological results** | **Age at vaccination initiation** | **Screened** | **Screen-positive and had biopsy** | **CIN2+** | **PPV** | | |
| --- | --- | --- | --- | --- | --- | --- | --- |
|  |  | n | n | n | **PPV for CIN2+, %**  **(95% CI)^b^** | **Crude RR** | **Adjusted^c^ RR** |
| High-grade cytology | |  |  |  |  |  |  |
|  | unvaccinated | 100 400 | 1909 | 1475 | 77.3 (75.3 to 79.1) | Reference | Reference |
|  | vaccinated at age 17-22 | 26 892 | 327 | 239 | 73.1 (67.9 to 77.8) | 0.95 (0.88 to 1.01) | 0.94 (0.87 to 1.01) |
|  | vaccinated at age <17 | 25 865 | 217 | 140 | 64.5 (57.8 to 70.9) | 0.83 (0.75 to 0.92) | 0.83 (0.74 to 0.92) |
| Low-grade cytology | |  |  |  |  |  |  |
|  | unvaccinated | 100 400 | 7610 | 2325 | 30.6 (29.5 to 31.6) | Reference | Reference |
|  | vaccinated at age 17-22 | 26 892 | 1614 | 377 | 23.4 (21.3 to 25.5) | 0.76 (0.70 to 0.84) | 0.76 (0.69 to 0.83) |
|  | vaccinated at age <17 | 25 865 | 1222 | 258 | 21.1 (18.9 to 23.5) | 0.69 (0.62 to 0.77) | 0.65 (0.58 to 0.74) |

1. Detection rate = No. of women confirmed as CIN2+ / No. of women screened * 100%
2. PPV = No. of women confirmed as CIN2+ / No. of women screen-positive and had histopathological diagnosis in biopsy * 100%
3. Adjusted for birth cohort

**Table S7 Detection rate, positive predictive value (PPV) of cytology and relative risks (RRs) for cervical intraepithelial neoplasia grade 1 (CIN1), in relation to age at vaccination initiation.**

| **Cytological results** | **Age at vaccination initiation** | **Screened** | **Screen-positive** | **CIN1** | **Detection rate of CIN1, %**  **(95% CI)^a^** | **PPV** | | |
| --- | --- | --- | --- | --- | --- | --- | --- | --- |
|  |  | n | n | n |  | **PPV for CIN1, %**  **(95% CI)^b^** | **Crude RR**  **(95% CI)** | **Adjusted^c^ RR**  **(95% CI)** |
| High-grade cytology | |  |  |  |  |  |  |  |
|  | unvaccinated | 100 400 | 2 110 | 278 | 0.3 (0.2 to 0.3) | 13.2 (11.8 to 14.7) | Reference | Reference |
|  | vaccinated at age 17-22 | 26 892 | 368 | 56 | 0.2 (0.2 to 0.3) | 15.2 (11.7 to 19.3) | 1.15 (0.89 to 1.51) | 1.19 (0.91 to 1.56) |
|  | vaccinated at age <17 | 25 865 | 244 | 37 | 0.1 (0.1 to 0.2) | 15.2 (10.9 to 20.3) | 1.15 (0.84 to 1.58) | 1.24 (0.88 to 1.73) |
| Low-grade cytology | |  |  |  |  |  |  |  |
|  | unvaccinated | 100 400 | 12 293 | 2 974 | 3.0 (2.9 to 3.1) | 24.2 (23.4 to 25.0) | Reference | Reference |
|  | vaccinated at age 17-22 | 26 892 | 2 940 | 616 | 2.3 (2.1 to 2.5) | 21.0 (19.5 to 22.5) | 0.87 (0.80 to 0.94) | 0.94 (0.86 to 1.01) |
|  | vaccinated at age <17 | 25 865 | 2 775 | 502 | 1.9 (1.8 to 2.1) | 18.1 (16.7 to 19.6) | 0.75 (0.69 to 0.81) | 0.90 (0.82 to 0.99) |

1. Detection rate = No. of women confirmed as CIN1 / No. of women screened * 100%
2. PPV = No. of women confirmed as CIN1 / No. of women screen-positive * 100%
3. Adjusted for birth cohort

**Table S8 Detection rate, positive predictive value (PPV) of cytology and relative risks (RRs) for cervical intraepithelial neoplasia grade 2 (CIN2), in relation to age at vaccination initiation.**

| **Cytological results** | **Age at vaccination initiation** | **Screened** | **Screen-positive** | **CIN2** | **Detection rate of CIN2, %**  **(95% CI)^a^** | **PPV** | | |
| --- | --- | --- | --- | --- | --- | --- | --- | --- |
|  |  | n | n | n |  | **PPV for CIN2, %**  **(95% CI)^b^** | **Crude RR**  **(95% CI)** | **Adjusted^c^ RR**  **(95% CI)** |
| High-grade cytology | |  |  |  |  |  |  |  |
|  | unvaccinated | 100 400 | 2 110 | 676 | 0.7 (0.6 to 0.7) | 32.0 (30.0 to 34.1) | Reference | Reference |
|  | vaccinated at age 17-22 | 26 892 | 368 | 107 | 0.4 (0.3 to 0.5) | 29.1 (24.5 to 34.0) | 0.91 (0.76 to 1.08) | 0.95 (0.80 to 1.13) |
|  | vaccinated at age <17 | 25 865 | 244 | 70 | 0.3 (0.2 to 0.3) | 28.7 (23.1 to 34.8) | 0.90 (0.73 to 1.10) | 0.99 (0.79 to 1.23) |
| Low-grade cytology | |  |  |  |  |  |  |  |
|  | unvaccinated | 100 400 | 12 293 | 1 692 | 1.7 (1.6 to 1.8) | 13.8 (13.2 to 14.4) | Reference | Reference |
|  | vaccinated at age 17-22 | 26 892 | 2 940 | 273 | 1.0 (0.9 to 1.0) | 9.3 (8.3 to 10.4) | 0.67 (0.60 to 0.76) | 0.75 (0.66 to 0.85) |
|  | vaccinated at age <17 | 25 865 | 2 775 | 183 | 0.7 (0.6 to 0.8) | 6.6 (5.7 to 7.6) | 0.48 (0.41 to 0.55) | 0.60 (0.52 to 0.71) |

1. Detection rate = No. of women confirmed as CIN2 / No. of women screened * 100%
2. PPV = No. of women confirmed as CIN2 / No. of women screen-positive * 100%
3. Adjusted for birth cohort

**Table S9 Detection rate, positive predictive value (PPV) of cytology and relative risks (RRs) for cervical intraepithelial neoplasia grade 3 or worse (CIN3+), in relation to age at vaccination initiation.**

| **Cytological results** | **Age at vaccination initiation** | **Screened** | **Screen-positive** | **CIN3+** | **Detection rate of CIN3+, %**  **(95% CI)^a^** | **PPV** | | |
| --- | --- | --- | --- | --- | --- | --- | --- | --- |
|  |  | n | n | n |  | **PPV for CIN3+, %**  **(95% CI)^b^** | **Crude RR**  **(95% CI)** | **Adjusted^c^ RR**  **(95% CI)** |
| High-grade cytology | |  |  |  |  |  |  |  |
|  | unvaccinated | 100 400 | 2 110 | 969 | 1.0 (0.9 to 1.0) | 45.9 (43.8 to 48.1) | Reference | Reference |
|  | vaccinated at age 17-22 | 26 892 | 368 | 152 | 0.6 (0.5 to 0.7) | 41.3 (36.2 to 46.5) | 0.90 (0.79 to 1.02) | 0.88 (0.77 to 1.00) |
|  | vaccinated at age <17 | 25 865 | 244 | 84 | 0.3 (0.3 to 0.4) | 34.4 (28.5 to 40.8) | 0.75 (0.63 to 0.90) | 0.72 (0.60 to 0.87) |
| Low-grade cytology | |  |  |  |  |  |  |  |
|  | unvaccinated | 100 400 | 12 293 | 951 | 0.9 (0.9 to 1.0) | 7.7 (7.3 to 8.2) | Reference | Reference |
|  | vaccinated at age 17-22 | 26 892 | 2 940 | 137 | 0.5 (0.4 to 0.6) | 4.7 (3.9 to 5.5) | 0.60 (0.51 to 0.72) | 0.58 (0.49 to 0.70) |
|  | vaccinated at age <17 | 25 865 | 2 775 | 94 | 0.4 (0.3 to 0.4) | 3.4 (2.7 to 4.1) | 0.44 (0.36 to 0.54) | 0.41 (0.33 to 0.51) |

1. Detection rate = No. of women confirmed as CIN3+ / No. of women screened * 100%
2. PPV = No. of women confirmed as CIN3+ / No. of women screen-positive * 100%
3. Adjusted for birth cohort
